# Impact of COVID-19 pandemic on time series of maternal mortality ratio in Bahia, Brazil: analysis of period 2011–2020

**DOI:** 10.1186/s12884-021-03899-y

**Published:** 2021-06-10

**Authors:** Rita de Cássia Oliveira de Carvalho-Sauer, Maria da Conceição N. Costa, Maria Gloria Teixeira, Estela Maria Ramos do Nascimento, Ediane Maria Filardi Silva, Mariana Luiza Almeida Barbosa, Géssica Rodrigues da Silva, Thaissa Piedade Santos, Enny S. Paixao

**Affiliations:** 1 Núcleo Regional de Saúde Leste, Bahia State Health Secretariat, Santo Antônio de Jesus, Bahia Brazil; 2 Institute of Collective Health, Federal University of Bahia, Salvador, Bahia Brazil; 3 Center of Data and Knowledge Integration for Health (CIDACS), Gonçalo Moniz Institute, Oswaldo Cruz Foundation, Salvador, Bahia Brazil; 4 Bahia State Health Secretariat, Salvador, Bahia Brazil; 5 Federal University of Recôncavo of Bahia, Santo Antônio de Jesus, Bahia Brazil; 6 London School of Hygiene and Tropical Medicine, London, UK

## Abstract

**Background:**

Most studies on the effects of SARS-CoV-2 infection have been conducted with adults and non-pregnant women. Thus, its impacts on maternal health are not yet fully established. This study aimed to verify the relationship between the maternal mortality ratio and the incidence of COVID-19 in the State of Bahia, Brazil, 2020.

**Methods:**

This time-series study used publicly available information in Brazil, to obtain data on maternal deaths and live births in Bahia, State, from January 1, 2011, to December 31, 2020. The time trend of Maternal Mortality Ratio (MMR) was analysed through polynomial regression, of order 6. Expected MMR, monthly (Jan-Dec) and annual values for 2020, were predicted by the additive Holt-Winters exponential smoothing algorithm, with 95% confidence interval, based on the time series of the MMR from 2011 to 2019, and the accuracy of the forecasts for 2020 was assessed by checking the smoothing coefficients and the mean errors. According to the statistical forecast, the MMR values ​​recorded in the year 2020 were compared to those expected.

**Results:**

In 2020, the annual MMR in Bahia, Brazil, was 78.23/100,000 live births, 59.46% higher than the expected ratio (49.06 [95% CI 38.70–59.90]). The increase in maternal mortality ratio relative to expected values was observed throughout the 2020 months; however, only after May, when the COVID-19 epidemic rose sharply, it exceeded the upper limit of the 95% CI of the monthly prediction. Of the 144 registered maternal deaths in 2020, 19 (13.19%) had COVID-19 mentioned as the cause of death.

**Conclusions:**

Our study revealed the increase in maternal mortality, and its temporal relationship with the incidence of COVID-19, in Bahia, Brazil, in 2020. The COVID-19 pandemic may be directly and indirectly related to this increase, which needs to be investigated. An urgent public health action is needed to prevent and reduce maternal deaths during this pandemic, in Brazil.

## Introduction

The emergence and global spread of the novel coronavirus, SARS-CoV-2, from December 2019, caused a respiratory disease (COVID-19) pandemic which was already affected until February 11, 2021 107,818,965 individuals and 2,362,704 deaths in the world [1]. The COVID 19 pandemic hit Brazil in February 2020, and 1 year later 9,524,640 cases and 231,534 deaths were reported, corresponding to 2,4% case fatality rate, and mortality rate of 110.2 deaths per 100,000 inhabitants [2], being on that date, the third country with the highest number of cases worldwide [1].

The clinical and epidemiological profile observed, at the beginning of the epidemic in China, showed that elderly (60 years old or more) with comorbidities such as diabetes, hypertension, heart disease, obesity, pneumopathies and immunosuppressed individuals were at higher risk for complications and deaths for COVID19, as has been observed in many countries affected by this pandemic [3–6]. At earlier stages of the epidemic, pregnant women without comorbidities were not considered at greater risk for COVID-19 and its related complications. However, with the greater spread of this disease, some authors began to describe the occurrence of severe forms, as well as abortion and deaths, even among pregnant women without comorbidities [7, 8].

These findings have been reported in many countries such as the United States, United Kingdom, France, Mexico and Brazil [2, 9–12], and contributed to subsidise PAHO, to issue an Alert in August 2020, encouraging member countries to redouble efforts to ensure access and continuity of prenatal care, with special attention to the early detection of signs, symptoms, and severity of clinical manifestations of COVID-19 [13]. It is known that during pregnancy the women present a relative immunodeficiency and it could worsen the clinical evolution of COVID 19 and lead to negative outcomes in the mothers and foetus [14].

Brazil is a middle-income country where the maternal mortality is still high [15] although it has been presenting a slow declining trend [16, 17]. Since the 1990 s, improvements in the living and health conditions of the population in this country, which includes women of reproductive age, have contributed to an important reduction in maternal deaths [18], but not enough to reach the Brazilian government target of less than 30 maternal deaths for every 100,000 live births [19]. Then, the COVID-19 pandemic may negatively influence the evolution of this indicator in Brazil.

Given the uncertainties about the possible impact of the COVID-19 on maternal mortality, this study aimed to verify the relationship between the maternal mortality ratio and the incidence of COVID-19 in the State of Bahia, Brazil, 2020.

## Methods

A retrospective time series study was carried out on maternal mortality during the COVID 19 pandemic in Bahia, Brazil, 2020. Maternal death was defined as established by the World Health Organization’s International Classification of Diseases (ICD-10) [20].

The website of the Bahia State Health Secretariat [21] was accessed to obtain the data for this study. Epidemiological bulletins [22] were the sources of data on the number of new cases of COVID-19 and population estimate, in Bahia. According to the month of occurrence, the number of maternal deaths and live births, from January 2011 to December 2020, were extracted from the public domain health open data, DATASUS [23, 24]. The DATASUS aggregates health data from different Brazilian official information systems. Data on maternal deaths come from the Mortality Information System (SIM), which in 2011 reached 96.1% nationwide coverage [25]. Data on live births come from the Live Birth Information System (SINASC), which in 2010 has coverage of 94,8% in Brazil, and most recent studies in large Brazilian cities point to 100% coverage [26, 27]. All maternal deaths occurred in 2020 were included in our analyses.

We estimated maternal mortality ratio (MMR) by dividing the number of maternal deaths by the number of live births, multiplied by 100,000, for each month and year [28]. Annual (from 2011 to 2020) and monthly (from January to December 2020) MMR were plotted on a time curve. The COVID-19 incidence in Bahia, per month in 2020, was calculated by the ratio between the number of new cases of this disease and the population estimate, followed by the multiplication by 100,000.

The time trend of MMR in the period from 2011 to 2020 was verified through polynomial regression analysis, of order 6. The additive Holt-Winters exponential smoothing model (ETS A, A, A), which considers the additive parameters error, trend and seasonality, was used to analyse the MMR time series 2011–2019 and predict the values for 2020, with a 95% confidence interval. Then, the real MMR values recorded in Bahia in 2020 were compared to those expected. The accuracy of the monthly and yearly MMR forecasts for 2020 was assessed by checking the Alpha, Beta and Gamma smoothing coefficients and the values of Scaled Mean Absolute Error (MASE), Symmetric Mean Percentage Absolute Error (sMAPE), Mean Absolute Error (MAE) and Root of the Mean Quadratic Simulation Error (RMSE).

As this study was carried out using public domain data, it was not necessary to submit it to the Research Ethics Committee.

## Results

**Fig. 1:**
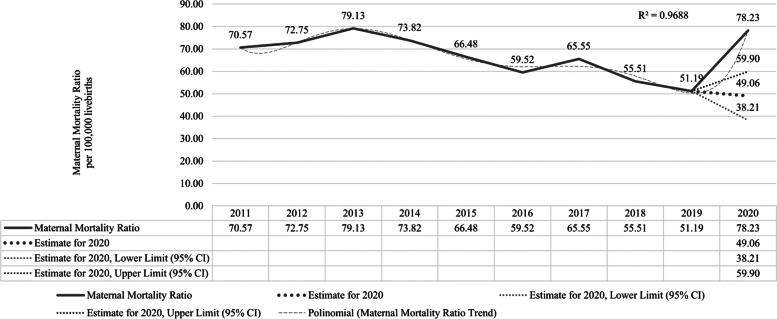
Maternal Mortality Ratio time series (per 100,000 live births) 2011–2019, and recorded* *versus* predicted^**^ values for 2020, with 95% confidence interval. Bahia, Brazil, 2011–2020. Source: Brazilian’s Mortality Information System (SIM/DATASUS) and Live Birth Information System (SINASC/DATASUS). *Data updated on February 04, 2021. ^**^ Values for 2020 predicted by Holt-Winters additive exponential smoothing, based on the 2011–2019 yearly MMR time series. Smoothing coeficients: Alpha = 0.75; Beta = 0.00; Gamma = 0.00. Forecast Accuracy: Scaled Mean Absolute Error (MASE) = 0.75; Symmetric Mean Percentage Absolute Error (sMAPE) = 0.07; Mean Absolute Error (MAE) = 4.56; Root of the Mean Quadratic Simulation Error (RMSE) = 5.53

Between 2011 and 2013, the MMR in Bahia increased by 12.1%. From 2013 to 2019, it was decreasing (-35.13%) over the years. However, in 2020, the MMR was 78.23/100,000 live births (lb.), an excess of 59,46% from the expected for this year (49.06 [95% CI 38.70–59.90]) (Fig. 1).

**Fig. 2:**
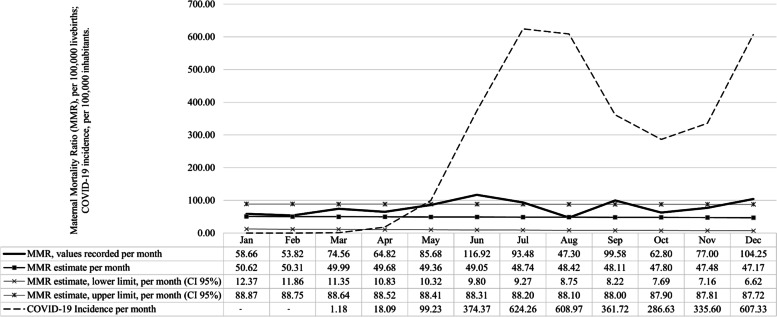
Maternal Mortality Ratio (MMR) per 100,000 live births, and the incidence of COVID-19 per 100,000 inhabitants, per month 2020*, and predicted^**^ MMR values with 95% confidence interval, for the same period. Bahia, Brazil, 2020. Source: Brazilian’s Mortality Information System (SIM/DATASUS) and Live Birth Information System (SINASC/DATASUS); COVID-19 Bahia Epidemiological Bulletins No. 01, 04, 35, 67, 98, 129, 160, 190, 221, 251 and 282/2020. *Data updated on February 04, 2021. ^**^Values for 2020 predicted by Holt-Winters additive exponential smoothing, based on the 2011–2019 monthly MMR time series. Smoothing coefficients: Alpha = 0.10; Beta = 0.00; Gamma = 0.00. Forecast Accuracy: Scaled Mean Absolute Error (MASE) = 0.57; Symmetric Mean Percentage Absolute Error (sMAPE) = 0.28; Mean Absolute Error (MAE) = 14.16; Root of the Mean Quadratic Simulation Error (RMSE) = 16.53

From January to April 2020, the predicted MMR values were not significantly different from the observed values for this period. Following a time pattern of increase and decrease like the COVID-19 incidence curve, in May the MMR increased and was close to the predicted maximum limit (Recorded MMR = 85.68/100,000 l.b.; Expected MMR = 49.36 [CI95% 10.32–88.41]). After that, the MMR exceeded the values expected for the period in June (Recorded MMR = 116.92/100,000 l.b.; Expected MMR = 49.05 [CI95% 9.80–88.31]), July (Recorded MMR = 93.48/100,000 l.b.; Expected MMR = 48.74 [CI95% 9.27–88.20]), September (Recorded MMR = 99.58/100,000 l.b.; Expected MMR = 48.11 [CI95% 8.22–88.00]) and December (Recorded MMR = 104.25/100,000 l.b.; Expected MMR = 47.17 [CI95% 6.62–87.72]) (Fig. 2).

From May to August, the COVID-19 pandemic rose sharply in Bahia, and its incidence per month reached 99.23, 374.37, 624.26, 608.97 / 100,000 inhabitants (inh.), respectively. A slight reduction in the incidence of COVID-19 was noted in September (361.72/100,000 inh.) and October (286.63/100,000 inh.), followed by a further increase in November (335.60/100,000 inh.) and December (607.33/100,000 inh.).

Of the 144 maternal death recorded in 2020, 19 (13.19%) were related to COVID-19 (Table 1) and occurred in the months April (01), May (02), June (05), July (06), September (02) and December (03). Of these, 15 death certificates contained mention of ICD-10 U071 (COVID-19, identified virus) among the causes of death, which means a SARS-CoV-2 infection diagnosed by laboratory examination, and 04 contained mention of ICD-10 U072 (COVID-19, unidentified virus), which represents diagnosis by clinical-epidemiological and imaging exams.

**Table 1:** Number of maternal deaths according to the classification of underlying causes group, by ICD-10 and year of occurrence, Bahia, Brazil, 2011–2020

**ICD-10**	**Underlying Causes Group**	**2011**	**2012**	**2013**	**2014**	**2015**	**2016**	**2017**	**2018**	**2019**	**2020**	
	**Year of occurrence**										**Total**	***With mention of COVID-19***^**a**^
A30-A49	Other bacterial diseases	-	-	1	-	-	-	-	-	-	-	*-*
B24	Human immunodeficiency virus [HIV] disease	3	-	1	2	1	2	4	1	-	1	*-*
D37-D48	Neoplasm of uncertain or unknown behavior	-	-	-	-	-	-	-	-	-	1	*-*
F50-F59	Behavioral syndromes associated with physiological disorders and physical factors	-	-	1	1	-	-	-	-	-	-	*-*
O00-O08	Pregnancy ending in abortion	12	12	4	11	11	10	16	10	4	11	*1*
O10-O16	Edema, proteinuria, and hypertensive disorders in pregnancy, delivery and the puerperium	25	30	33	38	35	23	25	25	30	32	*2*
O20-O29	Other maternal disorders predominantly related to pregnancy	2	4	4	1	4	3	7	1	2	9	*-*
O30-O48	Assistance provided to the mother for reasons related to the fetus and amniotic cavity and for possible problems related to delivery	12	9	9	9	6	13	7	9	8	18	*1*
O60-O75	Complications of labor and delivery	24	29	30	23	27	17	24	19	18	17	*1*
O85-O92	Complications related predominantly to the puerperium	14	18	23	26	14	12	14	12	11	12	*1*
O94-O99	Other obstetric conditions, not elsewhere classified	60	51	55	40	40	39	37	37	28	43	*13*
**Total**		**152**	**153**	**161**	**151**	**138**	**119**	**134**	**114**	**101**	**144**	***19***

Source: Brazilian’s Mortality Information System (SIM/DATASUS), data updated on February 04, 2021

^a^Number of maternal deaths, according to the underlying cause group of the ICD-10, with mention of COVID-19 among the causes attested in the death certificate

## Discussion

This study shows the rise in maternal mortality ratio in Bahia in 2020, insofar the officially recorded maternal deaths are far higher than the expected number for this year. A temporal relationship with COVID-19 pandemic was observed, since months in which the maternal mortality ratio exceeded the predicted value coincided with those with the highest incidence of COVID-19 in the state. The small number of maternal death certificates with COVID-19 diagnosed among the causes of death, which alone do not justify the excess of deaths observed in 2020, and leads us to consider the possibility of under diagnoses, since Brazil has not implemented universal testing of pregnant women [8] for COVID-19, and potential indirect effects of the pandemic. Maternal deaths attested due to not specified or ill-defined causes, related to ICD-10 O98.5—Other viral diseases complicating pregnancy, childbirth and the puerperium, for example, J18.9—Pneumonia, of unspecified aetiology or U04.9—Severe acute respiratory syndrome [SARS] unspecified, can mask undiagnosed cases of COVID-19.

Maternal deaths could result from maternal illness directly related to COVID-19 infection or an indirect effect of health service disruptions, and other indirect effects caused by the pandemic. First, maternal deaths directly related to COVID-19 in Brazil has been alarmed high [8]. However, it is also important to consider the possible indirect contributions of the pandemic in the occurrence of these deaths, as many pregnant women have stopped to attend antenatal appointments most likely because they did not feel protected against SARS-CoV-2 infection. Also, there may have been delay or resistance from some maternities with a lower level of complexity, to provide care to pregnant women with flu-like symptoms, or difficulty in the transportation of pregnant women to health units of a higher level of complexity located in other municipalities. In addition, some of these maternal deaths can still be under epidemiological investigation and analysis by the Epidemiological Surveillance Services and the State Maternal Mortality Studies Committee, so, the final cause of death can still be changed, and some deaths which COVID -19 has not been mentioned could emerge after evaluation. Lastly, the change in the organization of prenatal services during the pandemic, with a limited number of medical care provided to avoid crowding in the waiting rooms, may have caused delays in care, contributing to maternal deaths that could otherwise be avoided.

Maternal mortality is an indicator of access and quality of women’s health care [19], and this increase in maternal mortality showed in 2020 represents an unacceptable setback, that needs to be better clarified and faced. We understand that the public and private health sectors face many challenges in ensuring access and adequate care for all pregnant women. However, although the impact of the COVID-19 pandemic may affect many of them, it must be more remarkable for the poorest women, as occur already in the maternal mortality from other causes, to whom women of greater socioeconomic vulnerability are also more affected [18, 29, 30]. Therefore, the COVID-19 pandemic may exacerbate the social inequalities and injustices that already exist in Brazil [31, 32]. 

This study has limitations inherent to the use of i) secondary data; ii) relatively small number of maternal deaths, that makes the time series more susceptible to variations, and; iii) preliminary data on maternal deaths in 2020, due to possible delays in feeding official information systems. In addition, as time series studies correspond to a subtype of aggregate (or ecological) studies, they are vulnerable to ecological fallacy. Consequently, its results cannot be inferred for the individual level. Added to this the fact that it is a descriptive study, and thus a cause-effect relationship cannot be inferred from its results. Another limitation refers to the forecasting. In general, in the exponential smoothing of time series, the parameter ranges and initial values are arbitrary, and this can impair the accuracy of the forecast. In our study, it was possible to observe that the confidence interval for monthly forecasts was wider, indicating that there was less precision in these, when compared to the annual estimates. The model applied in our study use only the data from the series itself to project its forecasting, does not incorporate external variables, for example, environmental factors, or public policy interventions. Despite that, the Holt-Winters (ETS AAA) model is considered to be robust and has excellent performance for short-term forecasts [33, 34]. Notwithstanding these weaknesses, our study suggests devastating consequences for maternal mortality during the COVID-19 pandemic in Bahia, Brazil. It points to the need to conduct more research across the country during the pandemic to confirm these estimates and understand the long-term impacts of this disease on maternal health.

## Data Availability

The data that support the findings of this study are available on request from the first author (email contact: ritacarvalhosauer@gmail.com). The data are publicly available.

## Abbreviations

ICD: International Classification of Disease 10ª
MMR: Maternal mortality ratio
SIM: Mortality Information System
SINASC: Live Births Information System
inh.: Inhabitants
l.b.: Live births
